# Effects of novel coronavirus Omicron variant infection on pregnancy outcomes: a retrospective cohort study from Guangzhou

**DOI:** 10.3389/fmed.2023.1256080

**Published:** 2023-12-18

**Authors:** Huanshun Xiao, Cheng Chen, Shan Huang, Wenni Zhang, Shuangming Cai, Xiangling Hou, Yiping Luo, Yu Lin

**Affiliations:** ^1^Department of MICU, Guangdong Women and Children Hospital, Guangzhou, China; ^2^Department of Intensive Care Unit, Baiyun Branch of Nanfang Hospital, Southern Medical University, Guangzhou, China; ^3^Faculty of Science and Technology, BNU-HKBU United International College, Zhuhai, China

**Keywords:** pregnancy, novel coronavirus, Omicron, maternal and infant outcomes, retrospective cohort study

## Abstract

**Objective:**

Since 2022, Omicron has been circulating in China as a major variant of the novel coronavirus, but the effects of infection with Omicron variants on pregnant women and newborns are unknown. The purpose of this study was to determine the clinical characteristics of Omicron infection during pregnancy and its effect on pregnancy outcomes.

**Methods:**

This study retrospectively analyzed the data of 93 confirmed cases of novel coronavirus infection and 109 non-infected patients admitted to the isolation ward of Guangdong Maternal and Child Health Hospital from December 1, 2022 to January 31, 2023, and statistically analyzed the clinical features of Omicron variant infection during pregnancy and its impact on pregnancy outcomes. Further effects of underlying diseases on Omicron infection in pregnant women were analyzed.

**Results:**

The incubation period of COVID-19 infection was 0.99±0.86 days, 94.38% of patients had fever or other respiratory symptoms, the lymphocyte count in the infected group was lower than that in the uninfected group, and the lymphocyte count was further reduced in the patients with pregnancy complications or complications. Compared with the uninfected group, APTT and PT were prolonged, platelet count and fibrinogen were decreased in the infected group, all of which had statistical significance. COVID-19 infection during pregnancy increased the rate of cesarean section compared to uninfected pregnant patients, and COVID-19 infection in gestational diabetes resulted in a 4.19-fold increase in cesarean section rate. There was no statistically significant difference in gestational age between the two groups. The incidence of intrauterine distress, turbidity of amniotic fluid and neonatal respiratory distress were higher in the infection group. No positive cases of neonatal COVID-19 infection have been found.

**Conclusion:**

The patients infected with omicron during pregnancy often have febrile respiratory symptoms with lymphocyopenia, but the incidence of severe disease is low. Both Omicron infection and gestational diabetes further increase the incidence of cesarean section, and this study found no evidence of vertical transmission of Omicron.

## 1 Introduction

In December 2019, severe acute respiratory syndrome coronavirus type 2 (SARS-CoV-2) was detected in Wuhan, Hubei Province, China (1), and the disease was later named coronavirus disease (COVID-19) (2). Over the past 3 years, the novel coronavirus infection has spread around the world, causing a worldwide pandemic (3, 4). The severity of the disease ranges from mild asymptomatic infection to atypical pneumonia, acute respiratory distress syndrome, and multiple visceral failure (5), with high morbidity and mortality.

Large-scale studies have been reported on the impact of COVID-19 on the general population, but research and data on this group of pregnant women are still limited. Due to a variety of physiological changes during pregnancy, respiratory, circulatory, and immune system changes (6, 7), making pregnant women susceptible to various pathogens and prone to severe disease (8), during the epidemic, they are at high risk of contracting the new coronavirus (9). With the continuous understanding of the disease, infection with the novel coronavirus during pregnancy will have different degrees of impact on pregnant women and fetuses, affecting pregnancy outcomes (10–13).

Since the emergence of the pandemic, SARS-CoV-2 has been mutating constantly, with many variants emerging globally (14). There are five SARS-CoV-2 variants identified by the World Health Organization: Alpha (B.1.1.7), Beta (B.1.351), Gamma (P.1), Delta (B.1.617.2) and Omicron (B.1.1.529). Many mutations in variants, such as Delta and Alpha (15), are associated with immune evasion and higher infectivity, and there is uncertainty about the mutations in Omicron at this surface, and many mutations in the S protein of the Omicron variant may help the virus evade infection antibodies and other immune responses (16). The large number of mutations in Omicron increases the ability of the virus to enter cells and a higher risk of transmission (15, 16). Since 2022, Omicron has been circulating in China as the main variant (17), with a strong transmissibility with the adjustment of domestic epidemic prevention policies and the increasing number of infections. Some data from non-pregnant patients infected with Omicron have shown that it causes milder disease than those caused by previous variant infection (18, 19), and in clinical observations, pregnant patients have a similar situation, and with the general increase in awareness of the new coronavirus, 84.80% of people have received COVID-19 booster vaccination by June 2021 under the publicity and guidance of the Chinese government (20), studies have shown that COVID-19 vaccination has a promising effect on the prevention of SARS-CoV-2 infection, hospitalization, and ICU admission during pregnancy, with a reduction in preterm birth, and no increase in maternal comorbidities and complications, as well as adverse maternal and neonatal outcomes (21).

There are still many unknowns about the impact of the Omicron variant on pregnant women and newborns (22), and research reports are limited. This study summarizes the clinical data, maternal and infant data of pregnant women infected with the novel coronavirus variant admitted to our hospital and analyzes the clinical characteristics and pregnancy outcomes of patients, to provide reference for clinical treatment.

## 2 Clinical information and methodology

### 2.1 Study design and enrollment

This was a retrospective case–control study conducted at the Guangdong Women and Children Medical Hospital in which all adult women of different gestational ages tested positive for real-time reverse transcription polymerase chain reaction (RT-PCR) via nasal or throat swabs the infected group with confirmed SARS-CoV-2, and the non-infected group that tested negative. The infection group was divided into basic disease group and no disease group according to the underlying disease situation. Patients with unavoided miscarriage, requiring induction of labor, lack of data, and delivery of less than 34 weeks’ gestational age were excluded.

### 2.2 Data collection

Collect case data, including the patient’s age, body mass index (BMI), symptoms of COVID-19 infection (fever, nasal congestion, cough, sore throat, pulmonary signs, etc.), history of previous underlying medical conditions, and comorbidities during pregnancy (gestational diabetes, gestational hypertension, etc.), and record white blood cell count, lymphocyte count, hemoglobin, liver function [alanine aminotransferase (ALT), aspartate aminotransferase (AST), renal function blood urea nitrogen (BUN) and creatinine]. Coagulation function, recording fetal intrauterine distress, premature rupture of membranes, pregnancy outcomes (including delivery mode, gestational age, amniotic fluid traits); According to the needs of air defense, after the birth of the newborn, it will be transferred to the isolation area to take care of the newborn, record the newborn’s condition (including Apgar score, birth weight, SARS-cov-2 RT-PCR for two consecutive days, record the newborn’s body temperature, etc.) of data. Indications and number of pregnant women and newborns admitted to the ICU, etc. Preterm birth is defined as 37 weeks’ gestational age at the time of delivery <. Data is collected using developed forms and is reviewed and proofread by two colleagues. This study was reviewed and approved by the Medical Ethics Committee of Guangdong Maternal and Child Health Care Hospital. The medical ethics approval number is 202201345.

### 2.3 Statistical analysis

Statistical analysis was performed using SPSS 26. Continuous variables use the mean and standard deviation (SD) of the normal distribution, nonnormal distribution data use the median or quartile, and categorical variables use frequency and percentile. Data normality was checked using the Shapiro–Wilk, Kolmogorov–Smirnov test, and the *t*-test or Mann–Whitney-U was used the test compares continuous variables between infected and non-infected individuals, using the Pearson chi-square test or Fisher Exact testing compares categorical variables. An unadjusted odds ratio (OR) is calculated using a logistic regression model. *P* < 0.05 is considered statistically significant.

## 3 Results

### 3.1 Patient characteristics

A total of 198 patients were collected, including 93 in the infection group, and 4 patients who did not meet the inclusion criteria were excluded (2 were admitted for induction of labor due to fetal factors, 1 had missed miscarriage, 1 case data missing), 109 patients in the non-infected group, two groups of patients The basic situation and clinical features are shown in Table 1. There were no significant differences in the basic characteristics (including age, BMI, and underlying medical conditions) between the two groups.

**Table 1:** Patient characteristics and clinical features.

		Infected group (*n* = 89)	Non-infected group (*n* = 109)	*p*
Age (years) (mean ± standard deviation)		30.57 ± 3.50	31.53 ± 3.98	0.078
BMI (kg/m^2^) (*n*%)				0.352
	<25	36 (40.45%)	49 (45.0%)	
	25–29.9	46 (51.69%)	47 (43.1%)	
	30–34.9	6 (6.74%)	11 (10.1%)	
	35–39.9	1 (1.12%)	2 (1.8%)	
	≥40	0	0	
Prenatal underlying conditions	be	24 (26.96%)	30 (27.52%)	0.476
	not	65 (73.04%)	79 (72.48%)	
	Viral hepatitis B	8	8	0.689
	bladder cancer	2	1	0.867
	Hypothyroidism	5	6	0.972
	Abnormal liver function	3	4	0.91
	Hashimoto’s thyroiditis	1	1	0.885
	Subclinical hyperthyroidism	2	2	0.837
	Undifferentiated connective tissue disease	1	3	0.762
	Nephrupetosis	1	1	0.885
	arrhythmia	1	1	0.885
Pregnancy comorbid disease	be	24 (27%)	30 (27.5%)	0.522
	not	65 (73.0%)	79 (72.5%)	
	Gestational diabetes	20 (22.5%)	21 (19.3%)	0.580
	Hypertension during pregnancy	4 (4.5%)	9 (8.3%)	0.438
Vaccination status		83 (93.26%)	100 (91.74)	0.689
Past infection		0	0	-
COVID-19 symptoms (*n*%)	Incubation period (days)	0.99 ± 0.86	/	
	fever	84 (94.38%)	/	
	Sore throat	53 (59.55%)	/	
	Nasal congestion, runny nose	43 (50.56%)	/	
	cough	45 (48.31%)	/	
	headache	39 (43.82%)	/	
	Muscle soreness	38 (42.7%)	/	
	gasp	2 (2.25%)	/	
	dyspnea	2 (2.25%)	/	
	Asymptomatic infection	2 (2.25%)	/	
Treat	paracetamol	56 (62.92%)	/	
	No medication	33 (37.08%)	/	

The mean incubation period of COVID-19 infection in the infection group was 0.99 ± 0.86 days, and 97.75% of patients had influenza-like infection Symptoms, common symptoms include, fever (9 4.38%), sore throat (5 9.55%), nasal congestion, runny nose (50.56%), cough (4 8.31%), headache (4 3.82%), muscle aches (4 2.7%), shortness of breath, dyspnea (4.5%), asymptomatic 2 cases (2.25%), 6 Antipyretics were used in 2.92% of patients, the most commonly used drug was acetaminophen, and all patients did not use antiviral drugs. Both groups of patients received at least two doses of vaccine before pregnancy, with a vaccination rate of more than 90%, and no pregnancy was vaccinated. None of them had any previous infections.

### 3.2 Laboratory test results

The laboratory results of the two groups of patients are shown in Table 2. Compared with the non-infected group, 8 8.76% (*n* = 79) of patients in the infected group Lymphocyte count was reduced, and there was a statistically significant difference between the two groups (0.69 ± .034×10^^9^/L ratio (1.71 ± 0.51)×10^^9^/L, *p* < 0.001; The coagulation indexes of the two groups were within the normal range, but the infection group APTT and PT were prolonged, and the fibrinogen and platelet counts were reduced, and the differences between the two groups were statistically significant, 27.2 ± 2.81, respectively s ratio was 25.59 ± 1.75 s, 10.77 ± 0.53 s to 10.32 ± 0.52 s, 4.067 ± 0.79 g/L 4.57 ± 0.76 g/L, 194.99 ± 54.66)×10^^9^/L ratio (236.4 ± 69.16)×10^^9^/L, *p* is less than 0.05.

**Table 2:** Laboratory test results.

		Positive patients (*n* = 89)	Negative patients (*n* = 109)	*p*
Laboratory test results	White blood cell count	8.91 ± 2.29	9.69 ± 3.05	0.176
	Lymphocyte count	0.69 ± 0.034	1.71 ± 0.51	<0.001
	hemoglobin	112.09 ± 11.48	118.17 ± 13.07	0.001
	Platelet count	194.99 ± 54.66	236.4 ± 69.16	<0.001
	Serum albumin	33.41 ± 2.85	33.86 ± 4.2	0.041
	APTT	27.2 ± 2.81	25.59 ± 1.75	<0.001
	PT	10.77 ± 0.53	10.32 ± 0.52	<0.001
	fibrinogen	4.067 ± 0.79	4.57 ± 0.76	<0.001
	ALT	19.74 ± 10.7	22.27 ± 36.95	0.020
	AST	26.27 ± 11.39	24 ± 20.17	<0.001
	sCr	46.95 ± 9.87	46.24 ± 9.02	0.668
	BUN	6.21 ± 2.98	3.72 ± 1.85	0.030
	LDH	200.64 ± 22.99	204.34 ± 29.22	0.311
	PCT	0.099 ± 0.029	0.11 ± 0.03	0.482
Antiviral		0	/	
Anticoagulant therapy		0	0	
Thrombus incident		0	0	

The liver function and renal function indexes of the two groups were within the normal range, but the serum albumin and BUN increased in the infected group, which were statistically different compared with the non-infected group, respectively: serum albumin was 33.41 ± 2.85 g/L than 33.86 ± 4.2 g/L, *p* = 0.041, BUN6 21 ± 2.98umol/L ratio 3.72 ± 1.85 umol/L, *p* = 0.030. All patients were treated without anticoagulation and were followed up for 6 weeks with no thrombotic events.

### 3.3 Pregnancy and neonatal outcomes

The fetal heart rate of patients in the infected group was significantly faster than that in the non-infected group (164.46 ± 13.33 vs. 142.34 ± 5.01, *p* < 0.001), fetal intrauterine distress and amniotic fluid opacity increased significantly, respectively: 8 4.5% compared with the negative group (27.5%, *p* < 0.001) and (32.7% vs. 12.4%, *p* = 0.004); The cesarean section rate increased in the infected group (44.9% vs. 28.4%, *p* = 0.016). The difference was statistically significant. The gestational age of delivery was 37.51 ± 3.051 versus 36.74 ± 4.54, *p* = 0.251, respectively, and there was no significant difference in preterm birth rate between the two groups (infection group 11.2% was compared with the non-infected group 13.8%, *p* = 0.595), There was no difference in postpartum hemorrhage (2.25% versus 2.75%, *p* = 0.822). In addition, there were no statistically significant differences in threatened miscarriage and intrauterine/stillbirth.

Fifty-five births were delivered in 2 births in the infected group (3 twin births) and 101 births in the non-infected group (2 twin births) for a total of 103 newborns, with no difference in birth weight between the two groups. There were no significant differences in gestational age (38.28 ± 2.28 in the infected group compared with 38.12 ± 2.57 in the non-infected group, *p* = 0.719) and birth weight (3.304 ± 0.043 g vs. 3.419 ± 0.032 g, *p* = 0.094) between the two groups. Common neonatal complications include: neonatal asphyxia, low birth weight infants, neonatal pneumonia, etc., and the two cases in the infection group had birth Apgar scores of 1 min, 5 min, and 10 min, respectively 7–9-9 points, amniotic fluid III degree turbidity, improved after treatment; After delivery, all the children in the infection group were transferred to the neonatal department for isolation and observation, and there was no fever, tracheal intubation, unstable blood pressure, etc., and the new coronavirus antigen was tested for two consecutive days, and there were no positive results. One case of neonatal asphyxia occurred in the non-infected group, spontaneous delivery, maternal gestational diabetes, and poor glycaemic control during pregnancy Turbidity of amniotic fluid, Apgar score 7–8-8, admission to ICU treatment, diagnosis of aspiration pneumonia, oxygen and on He recovered and was discharged after antibiotic treatment. There were no deaths. See Table 3.

**Table 3:** Pregnancy and neonatal characteristics and outcomes.

		Positive patients (*n* = 89)	Negative patients (*n* = 109)	*p*
Gestational age (weeks)		37.51 ± 3.051	36.74 ± 4.54	0.251
Fetal heart rate		164.46 ± 13.33	142.34 ± 5.01	<0.001
Premature birth (< 37 weeks)		10 (11.2%)	15 (13.8%)	0.595
Threatened abortion		3 (3.37%)	0	0.103
Mode of delivery	Birth	12 (13.5%)	68 (62.4%)	<0.001
	Cesarean	40 (44.9%)	31 (28.4%)	0.016
Prelabour rupture of membranes		4 (4.5%)	25 (22.9%)	0.001
Intrauterine distress		75 (84.3%)	30 (27.5%)	<0.001
Perineal laceration		8 (9%)	5 (4.6%)	0.339
Cloudy amniotic fluid		17 (32.7%)	11 (12.4%)	0.004
Postpartum hemorrhage		2 (2.25%)	3 (2.75%)	1
Number of newborns		*n* = 55	*n* = 103	
Abnormal fetus		0	0	-
Gestational age at birth (weeks)		38.28 ± 2.28	38.12 ± 2.57	0.719
Birth weight (mean ± standard deviation) (kg)		3.304 ± 0.043	3.419 ± 0.032	0.094
Apgar score < 7		2 (3.6%)	1 (1.0%)	0.278
Neonatal complications		2 (3.6%)	1 (1.0%)	0.577
Newborns are positive for COVID-19 PCR		0	0	/
Newborns admitted to the ICU		0	1 (1.01%)	1

### 3.4 Infection group analysis based on the presence or absence of underlying disease subgroups

The 89 patients in the infection group were divided into 3 8 cases in the group with underlying diseases and 51 cases in the group without underlying diseases according to the underlying disease status The lymphocyte count in the group with underlying disease was further reduced by (0.55 ± 0.28)×10^^9^/L ratio (0.71 ± 0.36)×10^^9^/L, (*p* = 0.017), the difference was statistically significant. There were no differences in clinical symptoms, laboratory tests, mode of delivery, and neonatal outcomes of COVID-19 between groups. See Table 4.

**Table 4:** Subgroup analysis of infection groups.

		Associated with underlying disease (*n* = 38)	No underlying disease (*n* = 51)	*p*
Underlying disease	Gestational diabetes	20	/	
	Hypertension during pregnancy	6	/	
	Hypothyroidism	5	/	
	Hepatitis B	9	/	
	Abnormal liver function	3	/	
	Connective tissue disease	1	/	
	Hashimoto’s thyroiditis	1	/	
	Hyperthyroidism	2	/	
	Nephrupetosis	1	/	
	Bladder cancer	2	/	
	Arrhythmia	1	/	
COVID-19 Symptoms	Fever (°C)			0.779
	<37.3	2 (5.3%)	5 (9.8%)	
	37.3–38	11 (28.9%)	11 (21.6%)	
	38.1–39	21 (55.3%)	29 (56.9%)	
	39.1–41	4 (10.5%)	6 (11.8%)	
	>41	0	0	
	Sore throat	23 (60.5%)	30 (58.8%)	0.871
	Nasal congestion, runny nose	15 (39.5%)	28 (54.9%)	0.15
	Cough	16 (42.1%)	29 (56.9%)	0.168
	Headache	13 (34.2%)	26 (51.0%)	0.115
	Muscle soreness	13 (34.2%)	25 (49.0%)	0.162
	Gasp	1 (2.6%)	1 (2%)	0.834
	Dyspnea	1 (2.6%)	1 (2%)	0.834
	Pharyngeal hyperemia	20 (52.6%)	38 (74.5%)	0.032
	Crackles of the lungs	0	0	/
Heart rate (times/min) (mean ± standard deviation)		114.73 ± 17.63	114.25 ± 16.3	0.895
Gestational age (median)		37.58 ± 3.02	35.65 ± 5.44	0.054
Fetal heart rate		167.64 ± 13.07	162.42 ± 12.43	0.059
Laboratory tests	White blood cell count	8.32 ± 2.17	8.93 ± 2.95	0.285
Laboratory tests Premature birth (< 37 weeks)	Lymphocyte count	0.55 ± 0.28	0.71 ± 0.36	0.017
	Hemoglobin	109.85 ± 11.83	113.1 ± 10.36	0.172
	Platelet count	178.21 ± 60.28	201.18 ± 55.05	0.066
	Serum albumin	33.32 ± 2.06	33.98 ± 2.88	0.081
	APTT	26.63 ± 1.85	27.92 ± 3.17	0.028
	PT	10.85 ± 0.47	10.77 ± 0.54	0.507
	fibrinogen	4.06 ± 0.63	4.08 ± 0.87	0.859
	OLD	21.56 ± 13.82	17.51 ± 5.12	0.057
	AST	28.22 ± 12.9	24.78 ± 9.96	0.159
	sCr	47.76 ± 11.45	46.37 ± 8.57	0.512
	be	5 (21.7%)	5 (17.2%)	0.683
	not	18 (78.3%)	24 (82.8%)	
Mode of delivery	Birth	4 (10.5%)	9 (17.6%)	0.512
	Cesarean	19 (50.0%)	20 (39.2%)	
	Not in labor	15 (39.5%)	22 (43.1%)	
Prelabour rupture of membranes		0 (0%)	4 (3.7%)	0.129
Intrauterine distress		35 (92.1%)	40 (78.4%)	0.08
Postpartum hemorrhage		1 (2.63%)	2 (3.92%)	0.684
Neonatal complications		0	1	/
Neonatal coronavirus infection		0	0	/

In the infected group, univariate logistic regression analysis was used to determine the factors that increased the rate of cesarean section, and the incidence of cesarean section was increased in patients with gestational diabetes compared with those without diabetes 4 013-fold (OR = 4.013, 95% CI (1.368, 11.77), *p* = 0.011); In a multivariate multivariate logistic regression analysis that included age, BMI, gestational age, gestational hypertension, and gestational diabetes mellitus, the risk of cesarean section after COVID-19 infection in patients with gestational diabetes mellitus was still significantly associated with BMI < 25 (*p* = 0.011). See Table 5.

**Table 5:** Simple logistic regression model of cesarean section predictors for infection combination and underlying disease.

Variable	*p*	OR value	95% CI	
			Lower limit	Upper limit
Gestational diabetes	0.011	4.013	1.368	11.770
constant	0.031	0.581		

## 4 Discussion

This study showed that infection with the COVID-19 variant during pregnancy has a shorter incubation period, and the most common symptoms are fever, sore throat, nasal congestion, runny nose, cough and other flu-like symptoms. Most of these patients had fever (9 4.38%) and sore throat (59.55%), which were like the early epidemic among the Wuhan pregnancy cases reported by Chen et al. The most common symptoms were fever (75%) and cough (73%), 9 to 5 percent of patients showed similar results (23), except for fever The chance of symptoms is higher, unlike the 6 to 2.7% of pregnant women reported by Rasha Khoury et al. in the United States to be asymptomatic before or during hospitalization (24). Physiological changes in the respiratory, circulatory, and immune systems occur during pregnancy due to hormonal changes (8, 9), increasing the risk of respiratory virus infection during pregnancy and more serious clinical manifestations after infection (25). As the virus continues to mutate, virus virulence and pathogenicity change, and the Omicron variant decreases in its virulence and pathogenicity in causing lung infection or ARDS (26–28), clinical manifestations and characteristics also have different manifestations. Another reason for the reduction in clinical symptoms and the reduction in critical cases may be related to the high vaccination rate, with more than 90 percent of patients receiving more than two doses of vaccine before pregnancy, and like recent studies, vaccination has a high efficacy in preventing SARS-CoV-2 infection, hospitalization, and intensive care unit admission during pregnancy, and reducing preterm birth (20).

Compared with no infection, infection with the novel coronavirus during pregnancy was associated with a faster fetal heart rate, fetal intrauterine distress, increased occurrence of cloudy amniotic fluid, and an increased incidence of cesarean section (4 to 4.9%), but there was no difference in preterm birth rates. Previous studies have observed an increase in cesarean section rates in COVID-19-positive pregnant women (29–31), primarily due to clinical manifestations of severe disease following infection or exacerbations due to underlying medical conditions, pregnant women undergo cesarean section due to worsening respiratory symptoms (32, 33), and an increased rate of preterm birth may occur (34, 35). In our study, patients who did not have novel coronavirus pneumonia or who required respiratory support or were admitted to the ICU ward were classified according to the severity of symptoms (36). The patients enrolled in the study were mild and asymptomatic infections, with obvious symptoms and mild disease, which were consistent with the pathogenic characteristics of the variant Omicron (26–28) Infection with this variant is associated with a lower likelihood of severe illness in pregnant women (37, 38). This study showed that the indications for cesarean section are mainly fetal intrauterine distress, repeated high fever after infection with the virus, increased fetal heart rate, easy to be complicated by significantly increased fetal heart rate, and fetal intrauterine distress. However, after reasonable symptomatic treatment and other treatment, the symptoms were relieved, the condition did not worsen, and there was no difference in the rate of premature birth of patients.

From the results of laboratory tests, the lymphocyte counts of 8 8.76% of patients in the positive group is reduced, and it is also observed in the subgroup analysis of the underlying disease that the lymphocyte count of infected with the new coronavirus in the state of the underlying disease is lower than that of those without the underlying disease. Chen L et al. reported that lymphopenia was observed in 44% of pregnant women and COVID-19 clinical features (23); Guan WJ et al. reported that 83.2% of nonpregnant patients had lymphopenia, and severe patients were more obvious (39). Research by Lee J. et al. suggests that lymphopenia and its severity can be a reliable predictor of clinical outcome for COVID-19, with lower lymphocyte counts predicting more severe disease and increased need for therapeutic intervention (40). Whether lymphopenia is a predictor of COVID-19 in pregnancy requires further research and more data.

Hypercoagulable status during pregnancy is determined by increased prothrombotic factors (VII, VIII, X, XII, v-W factor F) and fibrinogen, decreased S protein and fibrinolysis, and detection of coagulation function Changes, fibrinogen and D-dimer increase, and APTT shortens (41, 42). The laboratory results of this study showed that the positive group had prolonged APTT and PT, and decreased fibrinogen and platelet counts, which were different compared with the negative group. The study of Pluta J. et al. showed that the most commonly observed changes in the coagulation system caused by COVID-19 were D-dimer, elevated fibrinogen, prolonged clotting time, no severe thrombocytopenia, and coagulation abnormalities DIC (disseminated intravascular coagulation) and SIC (coagulopathy due to sepsis) diagnostic criteria, described as COVID-19-related coagulopathy (43). COVID-19 variant infection has an impact on coagulation function, but they are all within the normal range, there is no difference in the risk of postpartum hemorrhage, and there is a risk of promoting thrombosis.

In this study, there was no anticoagulation during pregnancy and postpartum, and no thrombotic events occurred in either group. Thrombotic complications in patients with COVID-19 are pulmonary microvascular thrombosis (immunothrombosis) (44) or deep vein thrombosis (45). Changes in thrombotic and fibrinolytic may persist up to 3 months postpartum and then return to pre-pregnancy status (46, 47). Studies have not shown an increased incidence of VTE or immunothrombosis in patients with COVID-19, and no VTE events have been reported during treatment with prophylactic and therapeutic unfractionated heparin (UFH) or LMWH, Physical methods during pregnancy and postpartum and appropriate postpartum activity are important methods for preventing blood clots, and more clinical research is needed for routine medical prophylaxis. Given the risks, prophylactic anticoagulation in pregnant women with C covid-19 infection remains uncertain.

The most common cause of neonatal admission to NICU is respiratory distress, and risk factors for neonatal respiratory distress include prematurity, cloudy amniotic fluid, maternal diabetic chorioamnionitis, oligohydramnios, or structural lung abnormalities (48). In this study, the incidence of amniotic fluid opacity increased in the positive group, but there was no increase in complications such as neonatal respiratory distress, and there was no difference in NICU occupancy. Considering respiratory contagiousness, newborns were transferred to a neonatal isolation area for unified nursing care after birth, and COVID-19 PCR was tested daily by nasopharyngeal swab for two consecutive days to rule out vertical transmission or close horizontal transmission from the mother after delivery. No positive results for vertical transmission were observed. Previous studies have described positive rates of 2 to 4.4 percent of neonatal COVID-19 PCR tests at delivery (29, 49–51), but vary widely, PCR tests of COVID-19 such as amniotic fluid, cord blood, placenta, and breast milk have found no evidence of vertical transmission (52–54), and further research is needed.

Dicategorical univariate logistic regression analysis was used to screen for factors at risk of C-19 infection in pregnancy, and the susceptibility of both groups to COVID-19 infection. There was no difference, and pregnancy did not increase the risk of COVID-19. However, after infection with the virus, the incidence of cesarean section in patients with gestational diabetes increased by 4.01 times. Consistent with the findings of Helmut J. Kleinwechter et al. women with gestational diabetes have an increased risk of susceptibility to COVID-19 and severe disease, and adverse maternal outcomes are more common (55). Previous studies have also suggested that patients with underlying medical conditions (eg, hypertension, diabetes, obesity) are not only associated with disease severity, but also increase perinatal adverse outcomes (56, 57).

## 5 Conclusion

This study showed that most patients infected with the Omicron variant during pregnancy developed symptoms, and the rate of severe and critical cases decreased significantly. Viral infection can affect the coagulation system, and lymphopenia and severity may be related to disease and prognosis; Compared with the non-viral group, viral infections increased cesarean section rates, but there was no difference in the incidence of preterm birth, particularly in pregnant women with gestational diabetes, where infection led to a fourfold increase in the incidence of cesarean section. There was no difference in neonatal complications, there was no evidence of vertical infection, and separate care for mother and infant may help reduce the risk of neonatal infection.

The shortcomings of this study are that the admission cases are single-center data of the unit, and the inclusion of more data from other medical centers in the region or even in the region helps to reflect the conclusions more realistically, especially the conclusion that infection in gestational diabetes patients increases the incidence of cesarean section; Data on the symptoms and prognosis of the long coronavirus after infection during pregnancy are limited and longer follow-up is required.

## Data availability statement

The original contributions presented in the study are included in the article/supplementary material, further inquiries can be directed to the corresponding authors.

## Ethics statement

The studies involving humans were approved by this study was reviewed and approved by the Medical Ethics Committee of Guangdong Maternal and Child Health Care Hospital. The studies were conducted in accordance with the local legislation and institutional requirements. The participants provided their written informed consent to participate in this study.

## Author contributions

HX: Conceived the original idea, Provides data collection and data analysis, Writing—original draft. CC: Provides data collection and data analysis, Writing—original draft. SH: Contributed tables to the manuscript, Writing—review & editing. WZ: Contributed tables to the manuscript, Writing—review & editing. SC: Conceived the original idea, Writing—review & editing. XH: Contributed tables to the manuscript, Writing—review & editing. YLu: Conceived and designed this study, Writing—original draft. YLi: Conceived and designed this study, Writing—original draft. All authors contributed to the article and approved the submitted version.
